# *In vitro* controlled release of colon targeted mesalamine from compritol ATO 888 based matrix tablets using factorial design

**Published:** 2009

**Authors:** J.K. Patel, N.V. Patel, S.H. Shah

**Affiliations:** 1 *Nootan Pharmacy College, Visnagar, Gujarat - 384 315, India*; 2 *Department of Pharmaceutics, Anand Pharmacy College, Anand, Gujarat – 388 001, India*; 3 Department of Pharmaceutics, L. J. Institute of Pharmacy, Ahmedabad, Gujarat – 382 210, India

**Keywords:** Mesalamine, Compritol ATO 888, Pectin, Controlled drug delivery, Colon targeting, Factorial design

## Abstract

A controlled release matrix formulation for mesalamine was designed and developed to achieve a 24 h release profile. Using compritol 888 ATO (glyceryl behenate) as an inert matrix-forming agent to control the release of mesalamine, formulation granules containing the solid dispersions were investigated. Pectin, a polysaccharide, was used as bacterial dependent polymer for colon targeting. The matrix tablets for these formulations were prepared by direct compression and their *in vitro* release tests were carried out. A 3^2^ full factorial design was used for optimization by taking the amounts of glyceryl behenate (X_1_) and pectin (X_2_) as independent variables and percentage drug released at 2 (Q_2_), 16 (Q_16_) and 24 (Q_24_) h as dependent variables. Drug release from the matrix tablets formulations lasted for over 24 h. Images of the tablet surface and cross-section were characterized by scanning electron microscopy to show the formed pores and channels in the matrices. These may provide the release pathway for the inner embedded drugs. The co-mixing of polysaccharide pectin, into the waxy matrices played a meaningful role in targeting the tablets to colon. The drug release from the novel formulation may be attributed to the diffusion-controlled mechanism. The results of the full factorial design indicated that an optimum amount of compritol ATO 888 and a high amount of pectin favors the colon targeting and controlled release of mesalamine from dosage form.

## INTRODUCTION

Recently, a novel oral colon-specific drug delivery system (CDDS) has been developed as one of the site-specific drug delivery systems. This delivery system, by means of combination of one or more controlled release mechanisms, hardly releases drug in the upper part of the gastrointestinal (GI) tract, but rapidly releases drug in the colon following oral administration. The necessity and advantage of CDDS have been well recognized and reviewed recently(1–3). In view of the CDDS specifically delivering drug to the colon, a number of benefits would be realized in terms of improving safety and reducing toxicity when treating local or systemic chronic diseases. First, as for treating localized colonic diseases, i.e. ulcerative colitis, Crohn’s disease and constipation etc., the optimal drug delivery system, such as CDDS, should selectively deliver the drug to the colon, but not to the upper GI tract(1). As a result, the drug concentration will be significantly lessened in the upper GI tract, while increased considerably in the colon, resulting in the alleviation of GI side effects. Second, the colon is referred to as the optimal absorption site for proteins and polypeptides after oral administration, because of the existence of relatively low proteolytic enzyme activities and quite long transit time in the colon(2,3). CDDS can increase the bioavailability of proteins and polypeptides by releasing them nearly unchanged in the preferred colon and therefore protecting them against GI enzymatic degradation. Finally, CDDS would be advantageous when a delay in absorption is desirable from a therapeutical point of view, as for the treatment of diseases that have peak symptoms in the early morning and that exhibit circadian rhythms, such as nocturnal asthma, angina and rheumatoid arthritis(4,5).

Drug release behavior can be modified with the implication of solid dispersion technique. The diffusion and release properties of the insoluble active compounds are improved significantly to obtain enough solubility and release rate for the desired bioavailability(6–9). Currently, sustained or controlled release of water-soluble active ingredients can be achieved by utilizing the solid dispersion coating procedure with inert retarded materials. Due to their characteristics of waterinsoluble and non-swellable, waxy materials have major applications in sustained-release systems, especially for freely water-soluble drugs.

Glyceryl behenate, originally introduced as a lubricant for tablets, is a waxy material that has recently been used widely as a sustained-releas excipients(10,11). The use of glyceryl behenate as a hot-melt coating agent to prolong the release of theophylline has been investigated. The study has reported a satisfactory coating potential for this agent and a potential in sustaining the release of theophylline over an extended period of time.

Polysaccharides retain their integrity in the gastrointestinal tract because they are resistant to the digestive action of gastrointestinal enzymes. The matrices of polysaccharides are assumed to remain intact in the physiological environment of stomach and small intestine but once they reach in the colon, they are acted upon by the bacterial polysaccharidases that results in the degradation of the matrices. A large number of polysaccharides such as amylose, guar gum, pectin, chitosan, inulin, cyclodextrins, chondroitin sulphate, dextrans, dextrin and locust bean gum have been investigated for their use in colon targeted drug delivery systems(12,13).

Mesalamine is an active ingredient of agents used for the long-term maintenance therapy to prevent relapses of Crohn’s disease and ulcerative colitis. However, when mesalamine is administrated orally, a large amount of the drug is absorbed from the upper GI tract, and causes systemic side effects. Free mesalamine undergoes rapid and nearly complete systemic absorption from the proximal intestine depending on the concentration and local pH, followed by extensive metabolism(14). It is thus of tremendous importance to deliver mesalamine locally in order to reduce influences by systemic drug absorption causing adverse effects and drug loss decreasing the probability for a therapeutic success. Hence, selective delivery of mesalamine into the colon is desirable.

In this study, compritol 888 ATO was used as the waxy retardant material, pectin was used as a microbially triggered polysaccharide for colon targeting and mesalamine was chosen as the model of a freely water-soluble drug. The matrix tablets of mesalamine were prepared by direct compression of granules composed with solid dispersions of mesalamine, compritol 888 ATO and pectin. The release behaviors of mesalamine from the matrix tablets were studied *in vitro* and the imaging by scanning electron microscopy was used to inspect the porosity and morphology of the drug-released tablets. The effects of pectin polysaccharides on the release of mesalamine were also investigated. In the previous study, compression coated tablets which required special equipment and multistep procedure had been used for colon targeted delivery; while in this study novel time dependent polymer compritol ATO and bacteria dependent polymer pectin are used for the preparation of colon targeted matrix tablets utilizing solid dispersion granules.

## MATERIALS AND METHODS

### Materials

Mesalamine USP was a gift from Bec Chemicals Ltd. (India), Pectin obtained from L.V.G, (Ahmedabad, India). Ethyl cellulose, Methyl cellulose and Hydroxy propyl methyl cellulose (HPMC) were gifted by Colorcon Asia Pvt. Limited (Goa, India). All other materials were of reagent grade.

### Preparation of solid dispersions by hot fusion method

Compritol 888 ATO was melted in porcelain evaporating dish using a water bath at 55 °C. Mesalamine and pectin were added with continuous stirring (compritol 888 ATO, pectin and the drug were used in the required ratios for each preparation) to get a homogeneous dispersion. The molten mass with mesalamine present in its solid form was then allowed to cool down and solidify. Subsequently, the mass was ground, pulverized and passed through a 60-mesh sieve (less than 300 µm). The obtained powders were stored in desiccators at room temperature until use.

### Manufacture of compritol 888 ATO based matrix tablets

The solid dispersion was directly compressed using a 10-station rotary tablet machine (Cadmach, India) fitted with 9 mm diameter normal biconcave punches and die sets. Relatively constant tablet hardness was held around 8 kg for compression. The tablets, having luminous surfaces, were stored in a plastic container until use.

### Experimental design

A 3^2^ full factorial design was employed to systematically study the joint influence of the effect of independent variables X_1_ and X_2_ on the dependent variable. In this design, 2 factors were evaluated, each at 3 levels, and experimental trials were performed at all 9 possible combinations. A statistical model incorporating interactive and polynomial terms was used to evaluate the response.

Eq. 1 $$Y\;=\;b_{0}\;+\;b_{1}X_{1}\;+\;b_{2}X_{2}\;+\;b_{12}X_{1}X_{2}\;+\;b_{11}{X_{1}}^{2}\;+\;b_{22}{X_{2}}^{2}$$

where, Y is the dependent variable, b_0_ is the arithmetic mean response of the nine runs, and bi is the estimated coefficient for the factor Xi. The main effects (X_1_ and X_2_) represent the average result of changing one factor at a time from its low to high value. The interaction terms (X_1_X_2_) show how the response changes when 2 factors are simultaneously changed. The polynomial terms (X_1_^2^ and X_2_^2^) are included to investigate nonlinearity. The composition of the factorial design batches F1 to F9 are shown in Table 1.

**Table 1:** Composition of factorial design batches.

	Variable levels in coded form		Amount of drug released		
Batch Code	X_1_	X_2_	Q_02_ ± SD (%)	Q_16_ ± SD (%)	Q_24_ ± SD (%)
F1	-1	-1	18.2 ± 2.51	90.26 ± 1.22	99.99 ± 0.21
F2	-1	0	4.50 ± 0.12	85.26 ± 2.34	99.98 ± 0.62
F3	-1	1	0.00	80.26 ± 0.70	99.98 ± 0.54
F4	0	-1	14.2 ± 0.91	77.89 ± 0.92	99.98 ± 0.44
F5	0	0	3.80 ± 0.22	71.39 ± 1.64	98.99 ± 0.33
F6	0	1	0.00	66.72 ± 1.91	99.98 ± 1.21
F7	1	-1	12.3 ± 1.64	64.26 ± 2.63	90.26 ± 1.11
F8	1	0	3.30 ± 0.13	59.68 ± 0.70	85.26 ± 0.55
F9	1	1	0.00	53.67 ± 0.64	80.27 ± 1.02

**Coded values**	**Actual values**				
	X_1_	X_2_			

-1	75	25			
0	100	50			
1	150	75			

X_1_ indicates amount of compritol ATO 888 (mg) and X_2_ indicates amount. of pectin (mg) ± values represent the mean ± SD of 3 experiments. All batches contained 250 mg of mesalamine, 1% w/w talc and 1% w/w magnesium stearate

### Evaluation parameters

#### In vitro drug release study

The ability of the matrix tablets of mesalamine to remain intact and to release the active ingredient in the physiological environment of stomach, small intestine and colon was assessed by conducting *in vitro* drug release studies under conditions mimicking mouth to colon. The drug release studies (n=3) were carried out using USP dissolution rate test apparatus at 100 rpm and 37 ± 0.5 °C. Hydrochloric acid (900 ml of 0.1 M) was used as dissolution medium in the first 2 h of study as the average gastric emptying time was estimated 2 h. Five ml of the dissolution medium was withdrawn after 2 h to determine the drug release. The volume withdrawn was replaced with fresh media and this was accounted for during calculation of cumulative percentage drug release(15–17). The amount of drug release was measured using a double beam UV spectrophotometer (Lambda 2, Perkin– Elmer, USA) at maximum wavelengths of 301.5 nm. The dissolution media was replaced at the end of 2 h with Sorensen’s phosphate buffer, (pH 7.4, 900 ml) and drug release study was continued for another 3 h (i.e. total 5 h) as the average small intestine transit time is about 3 h. As before, samples were withdrawn at regular time intervals and correspondingly replaced with fresh media. The amount of drug release was measured using a double beam UV spectrophotometer (Lambda 2, Perkin– Elmer, USA) at maximum wavelengths of 334.5 nm.

### Preparation of rat caecal content medium

Before starting the experiments on animals, the experimental protocol was subjected to the scrutiny of the Institutional Animal Ethical Committee, and was approved by the same in time. The susceptibility of the matrix tablet to the enzymatic action of colonic bacteria was assessed by performing the drug release in medium contacting rat caecal contents. Caecal material was collected from male albino rats weighing 150-200 g, maintained on normal diet, but the caecal enzyme production was induced by giving orally 1 ml of 2% w/v dispersion of pectin for 7 days (administered directly into the stomach using Teflon tubing).

Thirty min before the commencement of drug release studies, four rats were killed and their abdomens were opened, the caecum was isolated, ligated at both ends, cut loose and immediately transferred into phosphate buffered saline (PBS) pH 6.8, previously bubbled with carbon dioxide (CO_2_). The caecal bags were opened and their contents were individually weighed, pooled and then suspended in PBS to give a final caecal dilution of 4% w/v. The dissolution study was continued in 100 ml of the above made rat caecal media after the 5th h. This was done with slight modification in the experimental set up of the USP dissolution rate test apparatus. A beaker of 150 ml capacity containing 100 ml of PBS (pH 6.8) with rat caecal content was placed suitably in the dissolution vessel having water maintained at 37 ± 0.5 °C which in turn was kept in the water bath of the apparatus. The study was continued from 5 h to 24 h and samples were withdrawn at regular intervals for analysis and replaced with fresh PBS media containing rat caecal material bubbled with CO_2_. The withdrawn samples were diluted with PBS and centrifuged. The supernatant was filtered through a bacteria proof filter and filtrate was analyzed for Mesalamine content using a double beam UV spectrophotometer (Lambda 2, Perkin– Elmer, USA) at maximum wavelengths of 299 nm.

### Scanning electron microscopy (SEM) analysis

The morphology of tablets may reflect the pathway and mechanism for drug release. So, SEM was used to image the tablet surfaces and cross-sections before or after the drug released. Samples were sputter coated with gold–platinum, and then imaged on an S-520 scanning electron microscope (Hitachi, Japan) at an accelerating voltage of 20 KV. SEM analysis of the tablet internal structure was also made after splitting the sample.

### Drug release kinetics

To study the release kinetics, data obtained from *in vitro* drug release studies were plotted in various kinetic models: zero order (Eq. 2) as cumulative amount of drug released vs. time, first order (Eq. 3) as log cumulative percentage of drug remaining vs. time, and Higuchi’s model (Eq. 4) as cumulative percentage of drug released vs. square root of time.

Eq. 2 $$C=C_{t}+k_{0}t$$

where, k_0_ is the zero order rate constant expressed in units of concentration/time and t is the time in h. A graph of concentration vs. time would yield a straight line with a slope equal to k_0_ and intercept the origin of the axes(18).

Eq. 3 $$\mathrm{Log}\;C=\mathrm{Log}\;C_{0}-\mathrm{kt}/2.303$$

where, C_0_ is the initial concentration of drug, k is the first order constant, and t is the time.

Eq. 4 $$Q=k\;t^{1/2}$$

where, k is the constant reflecting the design variables of the system and t is the time in h. Hence, drug release rate is proportional to the reciprocal of the square root of time(19).

To evaluate the drug release with changes in the surface area and the diameter of the particles/tablets, the data were also plotted using the Hixson-Crowell cube root law:

Eq. 5 $${Q_{0}}^{1/3}-{Q_{t}}^{1/3}\;=\;k_{HC}\;t$$

where, Q_t_ is the amount of drug released in time t,Q_0_ is the initial amount of the drug in the tablet, and kHC is the rate constant for the Hixson-Crowell rate equation(20), as the cube root of the percentage of drug remaining in the matrix vs. time.

### Mechanism of drug release

To evaluate the mechanism of drug release from matrix tablet, data for the first 60% of drug release were plotted in Korsmeyer-Peppas equation (Eq. 6) as log cumulative percentage of drug released vs. log time, and the exponent n was calculated through the slope of the straight line.

Eq. 6 $$M_{t}/M_{\infty }=\;k\;t^{n}$$

where, M_t_/M_0_ is the fractional solute release, t is the release time, k is a kinetic constant characteristic of the drug/polymer system, and n is an exponent that characterizes the mechanism of release of tracers(21). For cylindrical matrix tablets, if the exponent n=0.45, then the drug release mechanism is Fickian diffusion, and if 0.45< n <0.89, then it is non-Fickian or anomalous diffusion. An exponent value of 0.89 is indicative of Case-II Transport or typical zero order release(22).

### Stability study

Best formulation was exposed to three months stability study at 40 °C/75% relative humidity. These samples then again evaluated for evaluation parameters and *in vitro* drug release study(23).

### Data analysis

The response surface methodology is a collection of mathematical and statistical techniques used for modeling and analysis of problems in which a response of interest is influenced by several variables and the objectives is to optimize this response. The multiple regression analysis was done using DESIGN EXPERT 7.1.6 (STAT-EASE) demo version software. Analysis of data was carried out using ANOVA and the individual parameter was evaluated with F-test. Using the regression coefficient of factor, the polynomial equation for the each response is generated.

## RESULTS

### Full factorial design

The dissolution profile for 9 batches showed a variation (i.e., initial 2 h release ranging from 0% to 18% and drug released after 16 h ranging from 54% to 90 %). The data indicate that the release profile of the drug is strongly dependent on the selected independent variables. The fitted equations (full and reduced) relating the responses Q_2_,Q_16_ and Q_24_ to the transformed factor are shown in Table 2. The polynomial equations can be used to draw conclusions after considering the magnitude of the coefficient and whether it is negative or positive. Table 3 shows the results of the analysis of variance that was performed to identify insignificant factors(24). The high values of correlation coefficients for Q_2_,Q_16_ and Q_24_ indicate a good fit. The equations may be used to obtain estimates of the response, as a small error of variance was noticed in the replicates. The significance test for regression coefficients was performed by applying the student F-test. A coefficient is significant if the value of F is smaller than calculated F value.

**Table 2:** Calculations for testing the model in portions.

For Q_2_						
Regression	Df	SS	MS	r^2^	P	F_cal_ = 0.5510 F_table_ = 216
FM	5	377.36	75.47	0.9961	0.00080	DF = (1, 3)
RM	4	377.09	94.27	0.9954	0.00006	
Error						
FM	3	1.47	0.49			
RM	4	1.74	0.43			

**For Q_16_**						
**Regression**	**Df**	**SS**	**MS**	**r^2^**	**P**	**F_cal_ = 0.1776 F_table_ = 9.28**

FM	5	1186.7	237.3	0.9990	0.00010	DF = (3, 3)
RM	2	1186.5	593.3	0.9986	1.46E-09	
Error						
FM	3	1.14	0.38			
RM	6	1.35	0.22			

**For Q_24_**						
**Regression**	**Df**	**SS**	**MS**	**r^2^**	**P**	**F_cal_ = 4.77 F_table_ = 9.28**

FM	5	465.55	93.11	0.9815	0.00839	DF = (3, 3)
RM	2	423.76	211.9	0.8934	0.00121	
Error						
FM	3	8.76	2.92			
RM	6	50.5	8.42			

DF indicates degree of freedom; SS: sum of squares; MS: mean of squares; r^2^: regression coefficient; FM: full model; RM: reduced model

**Table 3:** Summary of results of regression analysis

Batch	Coefficient for Q_2_		Coefficient for Q_16_		Coefficient for Q_24_	
	Response (Q_2_)		Response (Q_16_)		Response (Q_24_)	
	FM*	RM*	FM	RM	FM	RM
b_0_	3.62	3.68	71.9	72.1	99.4	99.6
b_1_	-1.20	-1.20	-13.0	-13.0	-7.36	-7.36
b_2_	-7.46	-7.46	-5.29	-5.29	-1.67†	-----
b_12_	1.50	1.50	-0.15†	-----	-2.50†	-----
b_11_	0.37†	-----	0.23†	-----	-7.02	-7.02
b_22_	3.59	3.59	0.06†	-----	0.33†	-----

* FM: full model; RM: reduced model

† Response is insignificant at *P* = 0.05.

----- indicates term is omitted in reduced model

### Full and reduced model for Q_2_

**Fig. 1:**
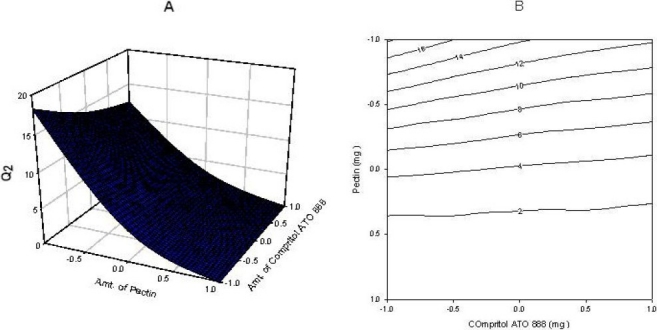
(A) Response surface plot showing the influence of compritol ATO 888 and pectin on Q_2_ and (B) Corresponding contour plot showing the relationship between various levels of 2 polymers.

The significance level of the coefficient b_11_ was found to be *P* = 0.5128, so it was omitted from the full model to generate a reduced model. The results of the statistical analysis are shown in Table 3. The coefficients b_0_, b_1_, b_12_ and b_22_ were found to be significant at *P*<0.05; hence, they were retained in the reduced model. The reduced model was tested in portions to determine whether the coefficient b11 contributed significant information to the prediction of Q_2_. The results of the model testing are shown in Table 2. The critical value of F for alpha = 0.05 is equal to 216 (DF = 1, 3). Since the calculated value (F = 0.5510) is less than the critical value (F = 216), it may be concluded that the interaction term_b11_ does not contribute significantly to the prediction of Q_2_ and can be omitted from the full model to generate the reduced model. A response surface plot was also prepared, as shown in Fig. 1.

### Full and reduced model for Q_16_

**Fig. 2:**
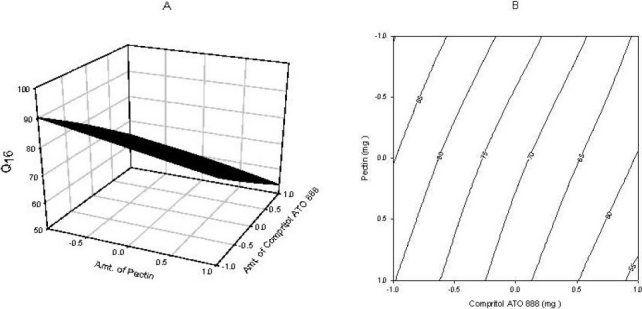
(A) Response surface plot showing the influence of compritol ATO 888 and pectin on Q_16_ and (B) Corresponding contour plot showing the relationship between various levels of 2 polymers.

The significance levels of the coefficients b12, b11 and b_22_ were found to be *P* = 0.66, 0.63 and 0.88 respectively. Therfore they were omitted from the full model to generate a reduced model. The results of the statistical analysis are shown in Table 3. The coefficients b_0_, b_1_ and b_2_, were found to be significant at *P*<0.05; hence, they were retained in the reduced model. The reduced model was tested in portions to determine whether the coefficients b_12_, b_11_ and b_22_ contribute significant information to the prediction of Q_16_. The results of the model testing are shown in Table 2. The critical value of F for alpha = 0.05 is equal to 9.28 (DF = 3, 3). Since the calculated value (F = 0.1776) is less than the critical value (F = 9.28), it may be concluded that the interaction terms b_12_, b_11_ and b_22_ do not contribute significantly to the prediction of Q_16_ and can be omitted from the full model to generate the reduced model. A response surface plot was also prepared, as shown in Fig. 2.

### Full and reduced model for Q_24_

**Fig. 3:**
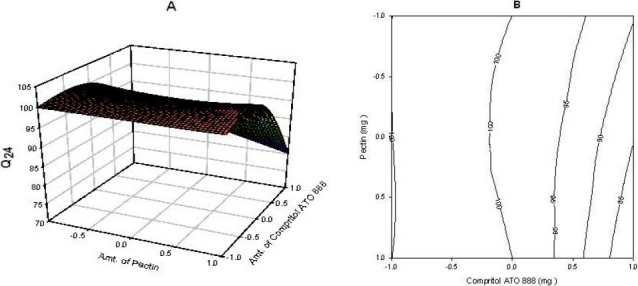
(A) Response surface plot showing the influence of compritol ATO 888 and pectin on Q_24_ and (B) Corresponding contour plot showing the relationship between various levels of 2 polymers.

The significance levels of the coefficients b_2_, b_12_, and b_22_ were found to be *P* = 0.09, 0.06 and 0.8, respectively. Therefore, they were omitted from the full model to generate a reduced model. The results of the statistical analysis are shown in Table 3. The coefficients b_0_ and b_1_ were found to be significant at *P*<0.05; hence, they were retained in the reduced model. The reduced model was tested in portions to determine whether the coefficients b_2_, b_12_, and b_22_ contribute significant information to the prediction of Q_24_. The results of the model testing are shown in Table 2. The critical value of F for alpha = 0.05 is equal to 9.28 (DF = 3, 3). Since the calculated value (F = 4.77) is less than the critical value (F = 9.28), it may be concluded that the interaction terms b_2_, b_12_, and b_22_ do not contribute significantly to the prediction of Q_24_ and can be omitted from the full model to generate the reduced model. A response surface plot was also prepared, as shown in Fig. 3.

### SEM imaging of the tablets before and after drug release

**Fig. 4:**
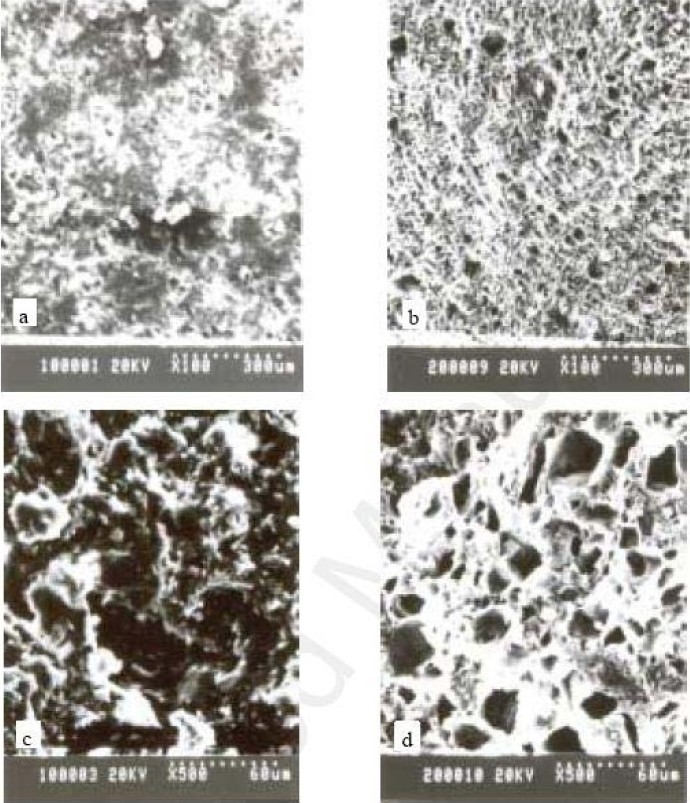
SEM micrographs of the surface and cross-sections of the matrix tablets compressed from the solid dispersions of mesalamine, pectin and compritol ATO 888 (a): surface of tablet before release test; (b): surface of the drug-released tablet; (c): transverse section of tablet before release test, (d): transverse section of the drug-released tablet.

Imaging technique of SEM can provide useful information about the surface characterization of the tablet surface(25). The microstructure of tablets transverse and longitudinal sections may reveal the pathway for drug release. Therefore, SEM was used to image the tablet surface and cross-sections before and after the drug release. Representative SEM images of surface and cross section of the matrix tablets are shown in Fig. 4. It can be seen from Fig. 4b, that the surface is full of pinholes which may be formed as a result of dissolution and diffusion of the drug particles at the surface of the matrices. This will allow the inner drug to release through the established mini-channels. The cross-section of the drug released tablet also showed a large number of cracks on its cut surface, which may reflect the multiplicity of porosity (Fig. 4c, d).

### Drug release

The zero order rate describes the systems where the drug release rate is independent of its concentration. The first order describes the release from systems where the release rate is concentration dependent. Higuchi’s model describes the release of drugs from an insoluble matrix as a square root of a time-dependent process based on Fickian diffusion.

The release constant was calculated from the slope of the appropriate plots, and the regression coefficient (r^2^) was determined (Table 4). It was found that the *in vitro* drug release of batch F6 was best explained by Higuchi’s equation, as the plots showed the highest linearity (r^2^ = 0.998), followed by zero order (r^2^ = 0.995). This explains why the drug diffuses at a comparatively slower rate as the distance for diffusion increases, which is referred to as square root kinetics (or Higuchi’s kinetics). However, drug release was also found to be very close to zero order kinetics, indicating that the drug release was nearly independent of the concentration. The dissolution data were also plotted in accordance with the Hixson-Crowell cube root law. The applicability of the formulation to the equation indicated a change in surface area and diameter of the tablets with the progressive dissolution of the matrix as a function of time.

**Table 4:** Drug release kinetics of batch F6

Zero Order		First Order		Higuchi		Korsmeyer-Peppas		Hixson-Crowell	
r^2^	K_0_ (% h^-1^)	r^2^	K_1_(h^-1^)	r^2^	K_H_ (h^-1/2^)	r^2^	n	r^2^	K_HC_ (h^-1/3^)
0.995	4.314	0.73	0.082	0.9979	8.629	0.901	0.57	0.995	-1.438

### Mechanism of drug release

The corresponding plot (log cumulative percent of drug release vs. time) for the korsmeyer-Peppas equation indicated a good linearity (r^2^ = 0.901). The release exponent n was 0.57, which appears to indicate a coupling of the diffusion and erosion mechanism, so-called anomalous diffusion, and may indicate that the drug release is controlled by more than one process. Reddy et al. observed similar results with a matrix tablet of nicorandil with an n value of 0.71(26) and Fassihi and Ritschel with a matrix tablet of theophylline with an n value of 0.7(27). Both these groups of researchers also considered the corresponding n values to indicate an anomalous release mechanism.

### Contour plots and response surface analysis

Two-dimensional contour plots and 3-D response surface plots, which are very useful to study the interaction effects of the factors on the responses, are shown in figures. These types of plots are useful in study of the effects of two factors on the response at one time. In all the presented figures, the third factor was kept at a constant level. All the relationships among the three variables are non-linear. As the amount of pectin is increased, the Q_2_ is decreased (Fig. 1). since pectin is a polysaccharide which is insoluble in gastric fluid and it is degraded by colonic microflora only when dosage form enters into colon. According to Fig. 3 it was concluded that as the amount of compritol ATO 888, time dependent polymer is increased, almost 100% drug release was achieved up to 24 h. So, higher level of pectin and medium level of compritol ATO 88 is beneficial for colon targeting drug delivery system. Response surface plots show the relationship between these factors even more clearly.

### Checkpoint analysis and stability study

Two checkpoint batches were prepared and evaluated for Q_2_, Q_16_ and Q_24_. Results indicate that measured release rates were as expected. When measured release rate values were compared with predicted release rate values using student’s t-test, the differences were found to be insignificant (*P*>0.5)(Table 5). Thus, it can be concluded that the statistical model is mathematically valid and the obtained mathematical equation is valid for predicting the amount of drug release. The factorial design batch (F6) was subjected to short term stability studies at 40 °C and 75% RH for 3 months. Studies indicate that no major change in evaluation parameters and *in vitro* release studies were observed.

**Table 5:** Checkpoint batches with their predicted and measured values of Q_2_, Q_16_ and Q_24_

Batch	X1	X2	Q_2_		Q_16_		Q_24_	
			Measured	Predicted	Measured	Predicted	Measured	Predicted
C1	-0.5	0.5	2.25 ± 0.22	1.19	76.32 ± 0.61	75.91	99.9 ± 1.72	101.20
C2	0.5	-0.5	8.45 ± 1.20	7.44	70.26 ± 0.22	68.20	94.6 ± 1.28	95.53

## DISCUSSION

Fig. 1–3 show the response surface plots and contour plots of the amount of glyceryl behenate and amount of pectin vs. Q_2_, Q_16_ and Q_24_ respectively. The plots were drawn using Sigma plot 11.0 software. The data demonstrate that both X_1_ and X_2_ affect the drug release. It was found that the addition 100mg of glyceryl behenate (X_1_) and 75 mg of pectin (X_2_) per tablet provided optimum controlled colonic drug delivery. The data clearly indicate that the dependent variables are strongly influenced by the independent variables. The high value of the correlation coefficient indicates a good fit. The polynomial equation can be used to draw a conclusion after considering the magnitude of coefficient and mathematical sign it carries (positive or negative).

From the data presented in Table 3 it is evident that the coefficient of factor X_1_ is negative for Q_2_,Q_16_ and Q_24_ indicating an antagonistic effect. It was anticipated that both of these matrices will be highly hydrophobic and would be expected to release the drug at a very slow rate, as indeed was found to be the case. The release rate of mesalamine from compritol 888 ATO based matrices was retarded effectively.

**Fig. 5:**
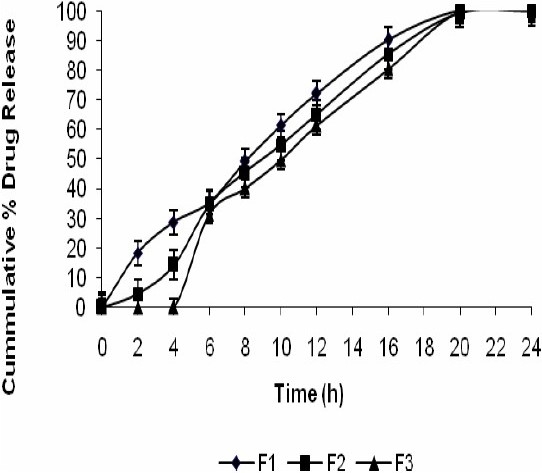
Release profile of mesalamine matrix tablets in 0.1 N HCL for 2 h, phosphate buffer (pH 7.4) for another 3 h, and phosphate buffer (pH 6.8) with rat caecal content till the end of 24 h.

**Fig. 6:**
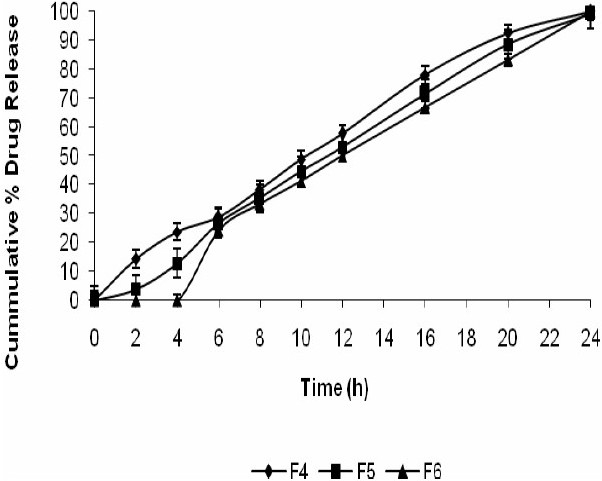
Release profile of mesalamine matrix tablets in 0.1 N HCL for 2 h, phosphate buffer (pH 7.4) for another 3 h, and phosphate buffer (pH 6.8) with rat caecal content till the end of 24 h.

**Fig. 7:**
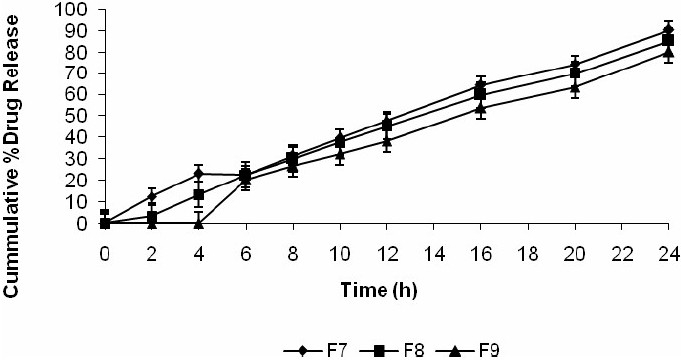
Release profile of mesalamine matrix tablets in 0.1 N HCL for 2 h, phosphate buffer (pH 7.4) for another 3 h, and phosphate buffer (pH 6.8) with rat caecal content till the end of 24 h.

As shown in Fig. 5–7, approximately 80-90% of the drug was released after 24 h for all the tablets prepared based upon the mechanical mixtures of mesalamine and pectin. However, for the solid dispersion compressed tablets prepared with higher concentrations of compritol ATO 888 less than 90% of mesalamine was released after 24 h and the tablets prepared with medium and low concentrations of compritol 888 ATO released nearly 99% of the drug. From the 16 h release data it was concluded that, increasing the concentration of compritol 888 ATO in the matrix will result in a further decrease in the drug release rate.

The slower release from the solid dispersion matrices is due to almost complete coating of the drug particles by compritol 888 ATO melted in the process of hot fusion. In this case, it is expected that the penetration of the dissolution medium into the matrix will be low compared with matrices prepared by mechanical mixtures, and hence, the dissolution and release of the drug occurs at a slower rate. Inspection of the appearance of the tablets, at the end of the dissolution test, revealed that all tablets containing compritol 888 ATO remained intact without any significant change in their shape. This indicates that the compritol 888 ATO used in the tablet formulation created an inert matrix It seems that the water-soluble active substance diffused across the waxy matrix.

From the data presented in Table 3 it was found that the coefficient of factor X_2_ is negative for Q_2_,Q_16_ and Q_24_ indicating an antagonistic effect. Matrix tablets formulated with low and medium levels of pectin failed to retain the tablets integrity during the dissolution studies in 0.1 M HCl and this problem could be due to the solubility of pectin in gastric fluid. But from Fig. 5–7 it was concluded that at higher percentage of pectin, the tablets could remain intact in the physiological environment of stomach and small intestine but once tablets enter into the colon, it is acted upon by polysaccharidases, which degrade the pectin and release the drug in the vicinity of bioenvironment of colon.

The controlled colonic drug delivery for the treatment of ulcerative colitis requirs that the dosage form to remain intact through its passage to colon and after reaching the colon it must release the mesalamine at constant rate up to 24 h. The results in Table 1 indicate that batch F6 fulfill the above criteria.

SEM images revealed the internal minitunnels with multiple voids resembling a microbore-network structure. These observations support our conclusion that the drug release is mainly due to diffusion through the channels formed in the matrix. These channels are formed by rapid dissolution of the watersoluble drug particles on the surface of the matrix. The aqueous medium would then penetrate into these channels for more dissolution of the drug presented in the deeper sites of the matrix.

## CONCLUSION

This study investigated the formulation of a controlled release colon targeted mesalamine tablet for the treatment of ulcerative colitis. The study showed that compritol 888 ATO is an appropriate waxy material that can be utilized as a matrix-forming agent to control the release of mesalamine. Pectin was used to deliver the drug specifically to the colon. A systematic study using a 3^2^ full factorial design reveled that the amount of added pectin and compritol ATO 888 had a significant effect on colon targeting and drug release up to 24 h. The optimum formula for colon targeting and controlled drug release was found to contain 75 mg of pectin and 100 mg of compritol ATO 888 for 250 mg of mesalamine. Drug release kinetics showed that the drug release was best explained by Higuchi’s equation, as indicated from the highest linearity (r^2^ = 0.998). However a close relationship was also noted with zero order kinetics (r^2^ = 0.995). Korsmeyer’s plots indicated a n value of 0.57, which was indicative of an anomalous diffusion mechanism or diffusion coupled with erosion. Hence, the drug release was controlled by more than one process. The prepared controlled-release matrix tablets would provide an extended 24 h duration of therapeutic effect of mesalamine with minimum potential for side effects.
